# Angiopoietin-2 and Biliary Diseases: Elevated Serum, but Not Bile Levels Are Associated with Cholangiocarcinoma

**DOI:** 10.1371/journal.pone.0097046

**Published:** 2014-05-13

**Authors:** Torsten Voigtländer, Sascha David, Kristina Thamm, Jerome Schlué, Jochen Metzger, Michael P. Manns, Tim O. Lankisch

**Affiliations:** 1 Department of Gastroenterology, Hepatology and Endocrinology, Hannover Medical School, Hannover, Germany; 2 Integrated Research and Treatment Center – Transplantation (IFB-Tx), Hannover Medical School, Hannover, Germany; 3 Department of Nephrology & Hypertension, Hannover Medical School, Hannover, Germany; 4 Institute of Pathology, Hannover Medical School, Hannover, Germany; 5 Mosaiques diagnostics GmbH, Hannover, Germany; Karolinska Institutet, Sweden

## Abstract

**Background:**

The diagnosis of cholangiocarcinoma (CC) is challenging especially in patients with primary sclerosing cholangitis (PSC) and often delayed due to the lack of reliable markers. Angiopoietin-2 (Angpt-2) has been employed as a biomarker of angiogenesis and might be involved in tumor neoangiogenesis.

**Aim:**

To evaluate the diagnostic potential of Angpt-2 as a biomarker to detect patients with CC.

**Methods:**

Bile and serum Angpt-2 levels were measured in patients with CC (n = 45), PSC (n = 74), CC complicating PSC (CC/PSC) (n = 11) and patients with bile duct stones (n = 37) in a cross sectional study. Diagnostic accuracy of Angpt-2 was compared to carbohydrate antigen 19-9 (CA19-9). Fluorescent immunohistochemistry from human CC liver tissue samples was performed to localize the origin of Angpt-2.

**Results:**

Serum Angpt-2 concentration was significantly elevated in patients with CC compared to control patients (p<0.05). Diagnostic accuracy of Angpt-2 as determined by receiver operating characteristic (ROC) analysis resulted in a higher area under the curve (AUC) value compared to CA19-9 (AUC: 0.85 versus 0.77; 95% confidence interval (CI): 0.74–0.93 versus 0.65–0.87, respectively). Angpt-2 was also detectable in bile, but was not associated with the presence of CC. Immunohistochemistry revealed a strong induction of Angpt-2 expression in the tumor vasculature.

**Conclusions:**

Circulating Angpt-2 in serum might be a promising protein candidate locally derived from the tumor vasculature in patients with CC. Measurement of Angpt-2 in serum may be useful for diagnosis and further clinical management of patients with CC.

## Introduction

Cholangiocarcinoma (CC) is the second most common primary liver cancer world-wide after hepatocellular carcinoma [1]. CC originates from the biliary epithelium and is classified by its localization in intrahepatic (ICC) and extrahepatic CC (ECC) [2], [3]. Growing evidence indicates that ICC and ECC differ regarding incidence and risk factors [1], [2]. In western countries, primary sclerosing cholangitis (PSC) is the main risk factor for later CC development [4]–[6]. PSC is complicated by CC in up to 13% of cases underlining the impact of this precancerous condition [6]. CC is regularly diagnosed at advanced stages due to the lack of early symptoms or reliable tumor biomarkers. Carbohydrate antigen (CA) 19-9 is often used as a biomarker for CC. However, CA 19-9 remains unspecific as it is also increased in benign biliary disorders such as cholestasis or cholangitis [7], [8]. In addition, the diagnostic accuracy of CA 19-9 in patients with PSC remains limited [9].

Consequently, there is a need for new diagnostic approaches (e.g. potent surrogate parameters for a reliable risk stratification) to improve the ability of an early detection and subsequently the clinical management of patients with CC.

The angiopoietin (Angpt)/Tie2 system consists of the transmembrane endothelial tyrosine kinase Tie2 and its antagonistic circulating ligands Angpt-1, and Angpt-2 [10]. The balance between the canonical agonist Angpt-1 and its competitive inhibitor, Angpt-2, regulates basal endothelial quiescence [11], [12]. Angpt-2 is strongly expressed in the vasculature of many tumors and it has been suggested that Angpt-2 may act synergistically with other cytokines to promote tumor-associated angiogenesis and tumor progression. It has been proposed that Angpt-2 might play a proangiogenic role in CC [13].

We therefore hypothesized that circulating levels of Angpt-2 might serve as a biomarker for the detection of CC. As the action of CC takes place at the biliary epithelium, we performed Angpt-2 analysis not only in serum, but also in bile in patients with CC, PSC, CC complicating PSC or bile duct stones and investigated Angpt-2 expression in human biopsies by immunohistochemistry.

## Patients and Methods

We included 167 patients (74 patients with PSC, 11 patients with CC/PSC, 45 patients with CC and 37 patients with bile duct stones) from the endoscopic unit of Hannover Medical School in a cross-sectional study during 2010 and 2012. The diagnosis of PSC was based on typical cholangiographic findings such as strictures or irregularity of intrahepatic and/or extrahepatic bile ducts after exclusion of secondary causes for sclerosing cholangitis. CC was confirmed histologically in 41 out of 45 patients. In four patients histology was not available, but clinical, laboratory, radiological and endoscopic retrograde cholangiography (ERC) findings were consistent with diagnosis of CC. The diagnosis of bile duct stones was based on ultrasound and/or endoscopic ultrasound and confirmed by ERC. Demographic data and laboratory values at day of ERC were recorded.

### Angpt-2 Measurement in Bile and Serum

Bile samples from patients were obtained during ERC as described before [14]. Serum samples were drawn whenever possible at day of ERC before endoscopic examination. Serum and bile Angpt-2 concentrations were measured by enzyme-linked immunosorbent assay (ELISA) according to the manufacturer’s instructions (R&D Systems, Minneapolis, MN, United States). The average value intra-assay coefficient of variation for Angpt-2 was 4.2%, and the average value inter-assay coefficient of variation was 7.4%. The sensitivity threshold was 8.29 pg/mL. All assays were performed in duplicate by investigators blinded to patients’ characteristics and outcome.

The study protocol was approved by the ethics commission of Hannover Medical School and is in accordance with the Declaration of Helsinki. Written informed consent was obtained from all patients.

### Fluorescent Immunohistochemistry from Human CC Samples

Slides from formalin-fixed, paraffin-embedded human CC samples were blocked for 60 min in 10% donkey serum, incubated with the primary antibody (polyclonal anti-human Angpt-2 AF 623, R&D Systems) in a dilution of 1∶50 overnight at 4°C, followed by fluorescence-conjugated affinity-purified secondary antibody labeling (60 min, 1∶500; Jackson ImmunoResearch, West Grove, PA), and mounted with ProLong Gold/4,6-diamidino-2-phenylindole (Life Technologies, Grand Island, NY). All images were taken by a Leica DMI 6000B (Leica Microscopy, Mannheim, Germany) immunofluorescence system at ×63 magnification. Of note, all images were obtained with the same laser power, gain, and offset conditions.

### Statistical Analysis

Group data are presented as number/percentages or median with interquartile range (IQR). All data were tested for normality (Shapiro-Wilk test, Kolmogorov-Smirnov test). Continuous data of the study groups were compared using Kruskal Wallis test. In case of statistical differences, Mann–Whitney or Wilcoxon test was used to compare groups. Receiver operating characteristics (ROC) curves were applied to determine diagnostic accuracy of the serological markers. Angpt-2 and CA 19-9 were included in a logistic regression model to detect an improvement in diagnostic accuracy of patients with CC. Correlation analysis was performed using Pearson or Spearman correlation as appropriate. P values <0.05 were considered statistically significant. The software used was the SPSS Statistical Package (version 19.0, SPSS Inc, USA), GraphPad Prism (version 6.01, GraphPad Inc, USA) and MedCalc Software (version 12.7.5.0, Belgium).

## Results

### Characteristics of the Study Cohort

Bile samples from 130 patients (68 PSC, 8 CC/PSC, 20 CC and 34 patients with bile duct stones) were obtained during ERC and corresponding serum samples at the same day were drawn from a total of 60 patients (27 PSC, 5 CC/PSC, 17 CC and 11 patients with bile duct stones). Additional 37 serum samples were analyzed (7 PSC, 2 CC/PSC, 25 CC and 3 patients with bile duct stones). Demographic characteristics and laboratory findings of the patient groups on day of ERC/day of blood collection are given in **Table 1**.

**Table 1:** Demographic characteristics, clinical features and laboratory findings of the patient groups on day of ERC/day of blood collection.

	All patients (n = 167)	Cholangiocarcinoma(CC) (n = 45)	Primary sclerosing cholangitis(PSC) (n = 74)	CC complicatingPSC (n = 11)	Bile duct stones(n = 37)	Referencevalue	p-value
**Gender**	M104, F63	M29, F16	M52, F22	M7, F4	M16, F21	−	0.05
**Age** (years)	51 (41–64)	64 (53–71)	42 (36–50)	52 (33–59)	60 (55–68)	−	0.001
**ECC**/**ICC**	−	37/8	−	7/4	−	−	−
**Metastatic disease** (y/n)	−	30/15	−	6/5	−	−	−
**Chemotherapy**(y/n)	−	21/24	−	4/7	−	−	−
**Laboratory values**							
ALT	52 (27–102)	54 (30–106)	54 (30–92)	51 (27–255)	40 (16–95)	<45 U/L	0.15
AST	51 (30–91)	58 (36–136)	52 (38–86)	71 (28–197)	31 (25–67)	<35 U/L	0.007
AP	253 (128–380)	336 (210–408)	244 (127–345)	263 (163–670)	134 (72–380)	40–129 U/L	0.006
GGT	198 (73–444)	364 (127–776)	166 (72–336)	114 (83–229)	159 (35–552)	<55 U/L	0.011
Bilirubin	18 (10–61)	48 (10–208)	16 (10–32)	41 (9–385)	13 (8–29)	<2–21 µmol/L	0.016
CRP	12 (4–41)	31 (5–80)	8 (3–28)	29 (6–36)	7 (3–31)	<8 mg/L	0.019
WBC	6.8 (5.5–9.8)	8.5 (6–11.8)	6.6 (5.2–9.7)	6.7 (5.8–7.2)	6.3 (5.4–8.4)	4.4 – 11.3/nL	0.058
LDH	191 (166–220)	191 (164–239)	181 (163–210)	180 (135–207)	206 (193–228)	<248 U/L	0.095
**Tumor markers**							
CA 19–9	41 (14–160)	142 (35–592)	29 (11–56)	43 (14–1820)	40 (13–91)	−	0.001
CEA	2 (2–4)	4 (2–6)	2 (1–3)	2 (2–3)	2 (2–4)	−	0.002
**Angiopoietin-2**							
Angpt-2 serum	4076 (2535–6881)	6497 (3789–9314)	2771 (2013–4559)	2746 (2618–4595)	2807 (1956–4723)	−	0.001
Angpt-2 bile	348 (190–604)	470 (238–1267)	331 (163–516)	304 (161–511)	358 (259–613)	−	0.227

**Abbreviations:** ECC, extrahepatic cholangiocarcinoma; ICC, intrahepatic cholangiocarcinoma; y, yes; n, no; M, male; F, female; ALT, alanine aminotransferase; AST, aspartate aminotransferase; AP, alkaline phosphatase; GGT, gamma-glutamyl transferase; CRP, C-reactive protein; WBC, white blood cells; LDH, lactate dehydrogenase; CA 19-9, carbohydrate antigen 19-9; CEA, carcinoembryonic antigen; angpt-2, angiopoietin-2.

### Serum Angpt-2 Levels are Significantly Elevated in Patients with CC

**Figure 1:**
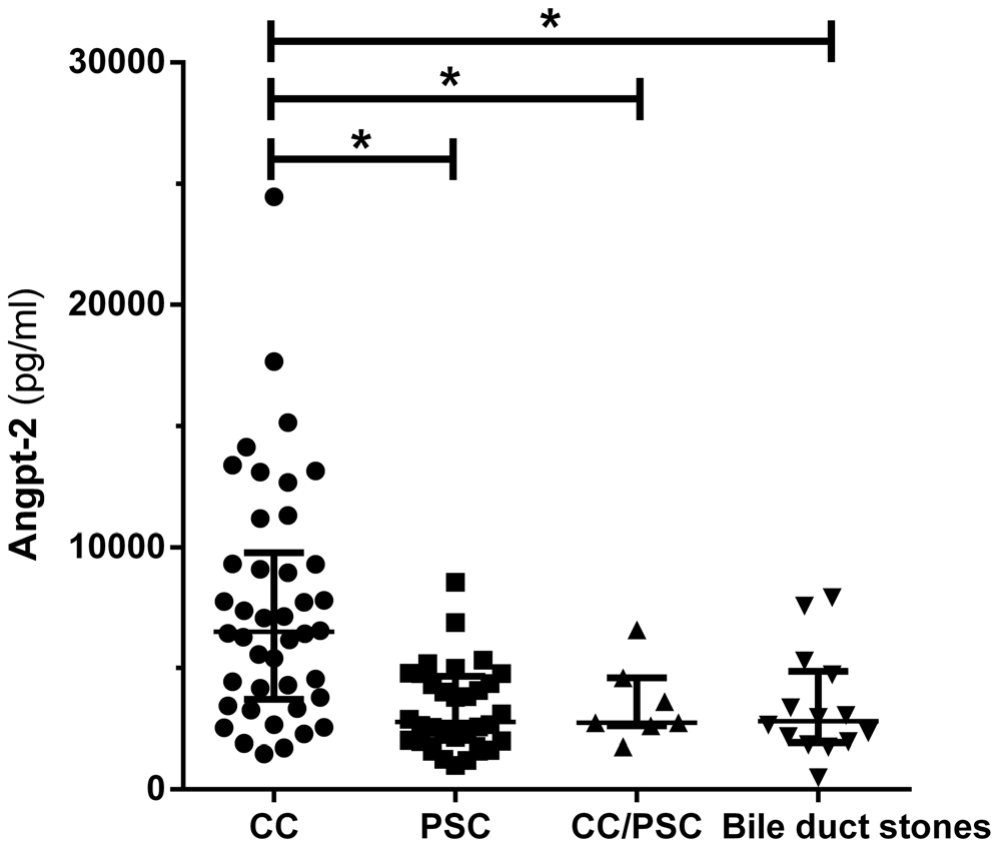
Serum Angpt-2 is elevated in patients with CC, (A). Angpt-2 was measured in patients with CC (n = 42), PSC (n = 34), CC/PSC (n = 7) and patients with bile duct stones (n = 14). Serum Angpt-2 was significantly elevated in patients with CC compared to control patients (p<0.01). Angpt-2 in bile is not associated with CC, (B). Bile concentration of Angpt-2 was measured in patients with CC (n = 20), PSC (n = 68), CC/PSC (n = 8) and patients with bile duct stones (n = 34). Four values (CC) are outside the scale of the Y-axis. No significant differences were detected among the groups.

Serum Angpt-2 concentrations among different groups are illustrated in **Figure 1A**. CC patients had the highest median serum concentration with 6497 (3789–9314) pg/ml (p = 0.01). PSC patients showed lower concentrations with 2771 (2013–4559) pg/ml. Patients with CC complicating PSC (2746 [2618–4595] pg/ml) and patients with bile duct stones (2807 [1956–4723] pg/ml) displayed lower concentrations, respectively. Regarding ECC and ICC, metastatic disease and pre-treatment with chemotherapy no significant differences in Angpt-2 serum concentration among CC patients were detected (p>0.05).

### Angpt-2 is Detectable in Bile but is not Associated with CC

Angpt-2 levels in bile were measured in 130 patients. Results are shown in **Figure 1B**. CC patients had the highest median Angpt-2 concentration in bile (470 [238–1267] pg/ml). In contrast, PSC patients (331 [163–516] pg/ml), CC/PSC patients (304 [161–511] pg/ml) and patients with bile duct stones (358 [259–613] pg/ml) showed lower concentrations. However, these differences did not reach statistical significance (p = 0.23). Correlation analysis between serum and bile Angpt-2 concentration showed no significant correlation (p>0.05).

### Diagnostic Accuracy of Serum Angpt-2 and CA 19–9

**Figure 2:**
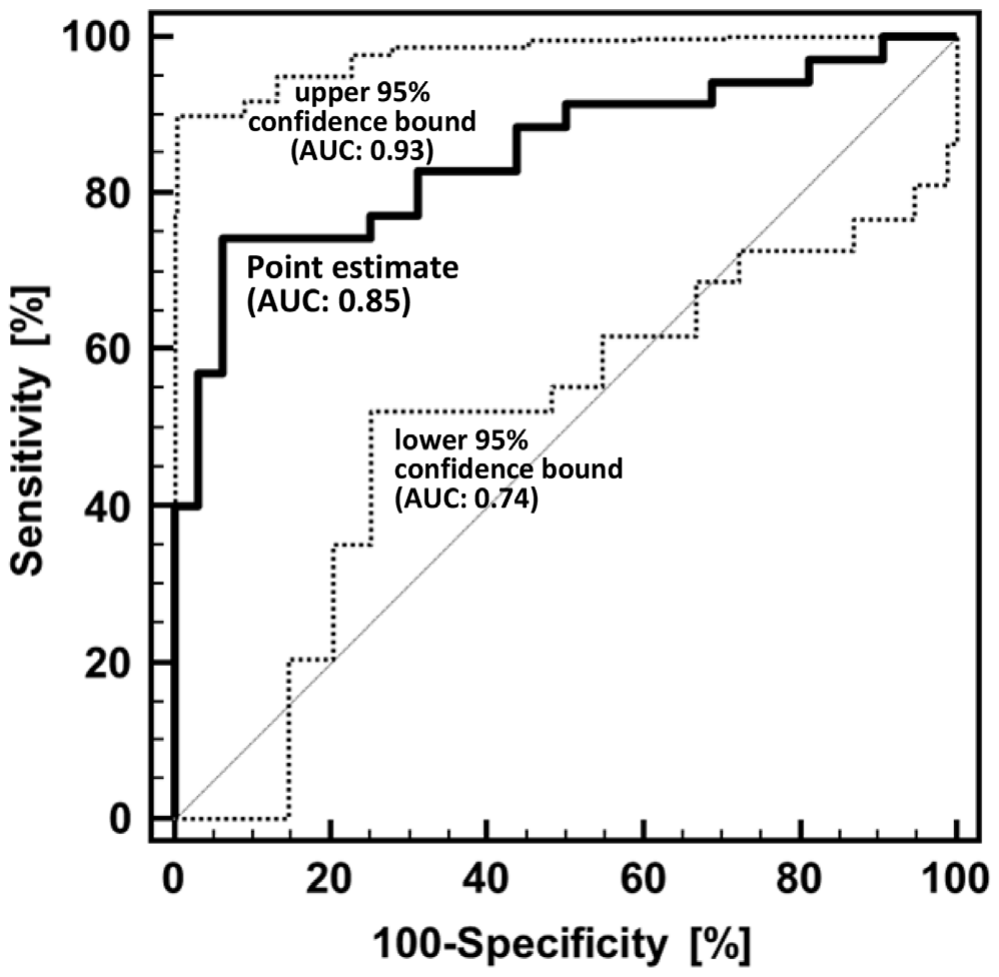
Receiver operating characteristic (ROC) curve analysis of serum Angpt-2 to diagnose CC. ROC analysis allows the evaluation of the binary classification variable (presence of CC = 1, absence of CC = 0) at different thresholds of Angpt-2 levels. The area under the curve (AUC) value for the point estimate (thick solid line) was calculated to be 0.85. The 95% confidence interval (CI) as the probability range in which the estimate is expected in 100 different cohorts of patients with equally distributed Angpt-2 levels ranges from an AUC of 0.74 in the worst case to 0.93 in the best case (thin dotted lines). The 95% CI is therefore a measure for the reliability of Angpt-2-based CC diagnosis. For testing the performance of Angpt-2 the departure of its AUC from 0.5 (a random classifier) was assessed by using a standard t test, which resulted in a p-value of <0.0001 for the point estimate.

In order to compare the diagnostic potential of serum Angpt-2 and CA 19-9 in patients with CC a receiver operating characteristic (ROC) curve analysis was performed. The area under the curve (AUC) for Angpt-2 was 0.85 (95% confidence interval 0.74–0.93, p = 0.01) (**Figure 2**). The sensitivity and specificity for Angpt-2 to diagnose CC was 74% and 94%, respectively (cut off value 5333 pg/ml). In contrast, the AUC for CA 19-9 in this patient cohort was 0.77 (95% confidence interval 0.65–0.87, p = 0.01). The sensitivity and specificity for CA 19-9 to diagnose CC was 53% and 82%, respectively (cut off value 130 U/ml). A combination of Angpt-2 and CA 19-9 in a logistic regression model did not improve the detection of CC patients (AUC 0.85, 95% confidence interval 0.74–0.93, p = 0.01).

### Angpt-2 Expression is Induced in the Tumor Vasculature

**Figure 3:**
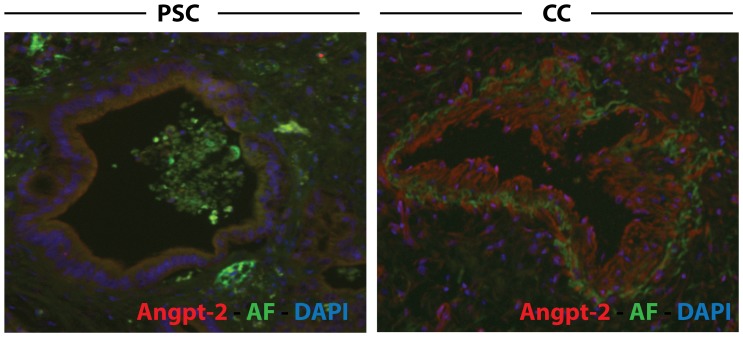
Angpt-2 expression in human CC samples. In human biopsies Angpt-2 expression is induced within CC vasculature. Fluorescent immunohistochemistry for Angiopoietin-2 (Angpt-2) showing an exemplary biopsy sample from individuals with primary sclerosis cholangitis (PSC, left panel) and cholangiocarcinoma (CC, right panel). Nuclear staining (DAPI) is shown in blue, tissue autofluorescence (AF) in green.

To investigate the origin of Angpt- 2 levels we performed an immunohistochemical analysis for Angpt-2 from liver tissue from patients with PSC or CC. Indeed, we observed a strong expression of Angpt-2 protein on the tissue level mostly limited to the tumor vasculature. **Figure 3** shows an exemplary comparison highlighting extensive Angpt-2 biosynthesis within the CC vasculature.

## Discussion

The detection of CC and differentiation from benign biliary strictures is challenging in clinical practice [15], [16]. Consequently, new diagnostic approaches are needed in order to improve the clinical management of patients with CC and tumor-predisposing conditions such as PSC. Proteomic analysis of bile and urine is a new promising technique as it analyzes a complete protein pattern in contrast to classical biochemical methods [17], [18]. However, these studies are complex, time-consuming and expensive and therefore supporting diagnostic tests are of special interest.

As the malignant transformation in CC takes place at the biliary epithelium analysis of bile might detect tumor-derived proteins in higher concentrations compared to serum specimens. To date, none of the single markers studied in bile has been implemented in clinical routine so far [19], [20].

Carcinogenesis in patients with CC includes a complex interaction between cancer-associated fibroblasts, remodeling of the extracellular matrix, immune cell migration and stimulation of proangiogenic factors [1], [16], [21]. Particularly, formation of new blood vessels is crucial for tumor maintenance, growth and metastasis as it supplies the neoplasia with essential nutrients and oxygen [22], [23]. Vascular endothelial growth factor (VEGF), VEGF receptors and nerve growth factor-β (NGF-β) are crucial mediators of pathological neoangiogenesis in the tumor environment and are also overexpressed in human CC samples favoring neoangiogenesis [24], [25]. Tang *et. al* analyzed surgically resected human CC samples and reported an association between tissue expression of VEGF, Angpt-2 and tumor microvessel density [13]. The role of the Angpt/Tie2 system, a second class of tyrosine kinase receptors, has not been fully evaluated in CC, but may play a role in tumor angiogenesis of other malignancies. Therefore, we hypothesized that Angpt-2 might also be involved in CC and may serve as a putative novel biomarker. This hypothesis is of high translational relevance as recently function-blocking antibodies against the Tie2 antagonist Angpt-2 have been developed and are currently investigated in ongoing clinical trials.

Initially, our study was designed to analyze circulating levels of Angpt-2 in patients’ sera, potential effects of the tumor on the local environment in terms of bile concentration of Angpt-2, and local Angpt-2 expression directly within the tumor by fluorescent immunohistochemistry from human tissue samples.

We found increased circulating levels of Angpt-2 in patients with CC compared to those with non-malignant pathologies. Our data indicate that Angpt-2 is useful as an additional novel marker. More importantly the data indicate a putative mechanistic involvement of Angpt-2 in tumor angiogenesis and might therefore represent a promising candidate for future therapeutic intervention. However, this aspect is clearly beyond the scope of the current project but warrants further investigation. We assume that elevated Angpt-2 in the serum reflects its potent - but most likely unspecific – mediator function in tumor neoangiogenesis as it has been demonstrated for other entities [26]–[29].

For the first time, we demonstrate that Angpt-2 is detectable in bile of CC patients. However, its pathophysiological role in bile is completely unknown. We hypothesize that in the sense of a “demand and supply” strategy the tumor cells might be able to produce Angpt-2 to maintain neoangiogensis at a high level. The detection of Angpt-2 in bile might be the result of tumor involvement of the large bile ducts and secretion of Angpt-2 from tumor vessels. A passive diffusion of Angpt-2 from blood vessels into the bile seems unlikely as no correlation was found for serum and bile Angpt-2 levels.

Taking the current Angpt/Tie2 knowledge in healthy conditions into account, one would assume that the tumor vasculature is the primary source of Angpt-2 biosynthesis as Angpt-2 is synthesized in the endothelium. With respect to this theoretical assumption and our earlier observation that Angpt-2 is also detectable in the bile we performed immunohistochemical stainings for Angpt-2. Of note, our histological data did show that it is indeed the vasculature where Angpt-2 is expressed in biopsies from human CC samples.

PSC patients are at special risk to develop CC and need accurate monitoring. In many patients, PSC is complicated by CC development despite regular surveillance [4]–[6]. Consequently, new diagnostic approaches are required. However, Angpt-2 measurement did not help to detect CC/PSC in our cohort.

Unfortunately, serum samples were not available for all patients which is a limitation of our study. Therefore we measured Angpt-2 in additional serum samples without concomitant bile specimens to provide sufficient data for statistical analysis.

In summary, we show that Angpt-2 is significantly elevated in patients with CC and has a higher AUC value in ROC analysis than CA 19-9 in our cohort. More importantly, Angpt-2 is expressed in the tumor vasculature and thus is of interest for the diagnosis and therapy in patients with CC.
